# Turn-taking in grooming interactions of sooty mangabeys (Cercocebus atys) in the wild

**DOI:** 10.1007/s10071-025-02040-2

**Published:** 2026-03-10

**Authors:** Pharisemène Tibesar, Catherine Crockford, Auriane Le Floch, Simone Pika

**Affiliations:** 1 Comparative BioCognition, Institute of Cognitive Science, Osnabrück University, Artilleriestrasse 34, 49076 Osnabrück, Germany; 2 The Ape Social Mind Lab, Institut Des Sciences Cognitives Marc Jeannerod, CNRS, 67 Boulevard PinelBron, 69675 Lyon, France; 3 Taï Chimpanzee Project, Centre Suisse de Recherches Scientifiques en Côte d’Ivoire, Abidjan, Ivory Coast; 4 Department of Human Behaviour, Ecology and Culture, Max Planck Institute for Evolutionary Anthropology, 04103 Leipzig, Germany; 5 Institute of Biology, University of Neuchâtel, Neuchâtel, Switzerland; 6 Taï Monkey Project, Centre Suisse de Recherches Scientifique en Côte d’Ivoire, Abidjan, Ivory Coast

**Keywords:** Social interaction, Participation framework, Temporal relationships, Adjacency pairs, Evolution of language, Gestures

## Abstract

Language is widely regarded as a uniquely human trait, rooted in mechanisms of social interaction such as conversational turn-taking. It has been proposed that turn-taking may not be exclusive to humans but may represent an ancestral trait shared across primates and beyond. Early studies on great apes indicated a specialization in gestural forms of turn-taking, whereas monkeys appeared to rely more heavily on vocal exchanges. However, this ape–monkey difference may simply be due to a lack of research. To bridge this gap, the present study investigated turn-taking behaviors during grooming in an Afro-Eurasian monkey species, sooty mangabeys (*Cercocebus atys)*. We addressed the following three key questions: (1) What turn-taking patterns, if any, characterize grooming interactions in sooty mangabeys? (2) Do sooty mangabeys preferentially use vocal turn-taking? (3) Which elements characterizing turn-taking patterns of sooty mangabeys are shared with human conversational turn-taking? We collected 105.3 h of video footage from 22 adult female sooty mangabeys in Taï National Park (between May 2022 and August 2023), yielding 428 female–female grooming videos, of which 71 were coded. An additional 595 grooming interactions were described directly in the field. Results showed that exchanges involving both actions and signals were the most frequent (e.g., a present gesture followed by grooming). The most common type of signal exchange was gestural turn-taking, a form previously reported only in great apes. The study identified key elements of conversational turn-taking: rapid responses, adjacency pair-like sequences, and participation frameworks. These findings suggest that the fundamental structural principles of human conversation likely predate our species, offering valuable insight into the evolutionary history of language.

**Supplementary Information:**

The online version contains supplementary material available at 10.1007/s10071-025-02040-2.

## Introduction

Although it has intrigued scientists for centuries, the evolution of language still remains a mystery (Fitch 2002; Hewes et al. 1973; Knight et al. 2000). While the specific processes underlying language evolution are not fully understood (Bickerton 2016; Chomsky 2010), it is, however, widely accepted that language emerged relatively recently in human history (Diller & Cann 2009; Miyagawa et al. 2025; Nóbrega & Miyagawa 2015). This temporal proximity supports the hypothesis that the entire cognitive apparatus required for language could have developed based on already existing mechanisms and abilities (Hewes et al. 1973; Jackendoff 1999; Levinson & Holler 2014). Currently, the two predominant hypotheses suggest evolutionary precursors in either vocal (Hockett 1960; Lemasson et al. 2011; Locke & Hauser 1999) or gestural means (Arbib et al. 2008; Call & Tomasello 2020; Corballis 1999; Hewes et al. 1973). Recently, however, Levinson (2016a, 2019) proposed that the evolution of language is deeply intertwined with a range of interactional abilities, collectively referred to as the interaction engine. This hypothesis suggests that human communication is evolutionarily stratified, with language emerging by using pre-existing abilities, such as joint attention, mutual gaze, and cooperative turn-taking. The latter stands out as representing one of the most ancient layers (Levinson & Holler 2014; Pika et al. 2018), with scholars suggesting a possible phylogenetic continuity between human social action during conversations and communicative interactions of other primate species and beyond (Abreu & Pika 2022; Levinson 2016b; Pika et al. 2018).

Turn-taking refers to the structured organization of participation in various activities, in which individuals take turns (Sacks et al. 1974). Turn-taking plays a crucial role across diverse contexts such as allocating political office, games, traffic management, and debates (Helbing et al. 2005; Natalia et al. 2019; Smith 2020). Turn-taking may involve either actions (e.g., children building a toy tower by alternating placing the blocks) or signals (e.g., speech) or a combination. Particular research attention has been dedicated to conversational turn-taking, which is part of the ‘speech exchange systems’ in humans (Sacks et al. 1974). Conversational turn-taking refers to the cooperative, rapid, and reciprocal exchange of signals, with turns being allocated between at least two participants (Sacks et al. 1974). While significant overlaps and gaps between turns are rare, conversational turn-taking is highly flexible, and interactants use specific turn-allocation techniques (e.g., vocal cues such as “go ahead” or pausing to signal the end of a turn), as well as repair mechanisms (e.g., clarification requests or self-corrections to address turn-taking errors or violations) (Sacks et al. 1974).

Turn-taking has been proposed to have preceded language in phylogeny due to several reasons (Levinson 2016b). First, although the over 6,700 languages so far described show a high degree of diversity (Comrie 2003; Pereltsvaig 2020), the involved turn-taking structures seem to exhibit strong universality (Stivers et al. 2009). Second, a study across 10 different languages revealed a consistent pattern in response offsets, with a unimodal peak occurring within 200 ms after a question was posed (Stivers et al. 2009). This is extremely fast, considering that it takes between 600 and 1000 ms to plan and produce a single-word response in a conversation (Bates et al. 2003; Indefrey and Levelt 2004). Hence, the processes of speech comprehension and production must overlap significantly to achieve such rapid response timing (Levinson 2016b; Levinson & Holler 2014). Third, turn-taking emerges very early in infancy, with babies at the age of three months already starting to coordinate smiles, gazes, and facial expressions with their caregivers (e.g., Bates et al. 1975; Bateson 1975; Gratier et al. 2015). Fourth, evidence suggested that some form of turn-taking may be present in several nonhuman primate species (hereafter primates) (Fröhlich et al. 2016a, b; Lemasson et al. 2011; Levinson 2016b; Takahashi et al. 2013). This includes great apes such as bonobos (*Pan paniscus; *Fröhlich et al. 2016a; Levréro et al. 2019; Rossano 2013) and chimpanzees (*Pan troglodytes; *Fröhlich et al. 2016a); smaller apes such as lar gibbons (*Hylobates lar; *Terleph et al. 2022) and Mueller’s gibbons (*Hylobates muelleri; *Inoue et al. 2013); Afro-Eurasian monkeys such as Campbell’s monkeys (*Cercopithecus campbelli; *Lemasson et al. 2011) and Diana monkeys (*Cercopithecus diana; *Candiotti et al. 2012); and Neotropical monkeys such as common marmosets (*Callithrix jacchus; *Takahashi et al. 2013) and spider monkeys (*Ateles geoffroyi; *Briseño-Jaramillo et al. 2018). If this hypothesis is correct, investigating turn-taking can offer critical insights into the underlying structures and mechanisms that existed prior to the emergence of human language and contributed to its evolution (Levinson 2016b). One useful approach to this investigation is the comparative approach, which examines the behavior of other animals to then infer the abilities of our common ancestor and gain an understanding of the adaptations that enabled the evolution of behaviors, such as language (Hodos & Campbell 1969; Pika 2008; Zuberbühler 2005). Systematic and informed cross-species comparisons further refine this approach by enabling the reconstruction of evolutionary trajectories across lineages (Fitch 2002; MacLean et al. 2012; Pika et al. 2018). Comparisons with distantly related species provide complementary insights, as they allow the identification of patterns due to analogies, in which similar traits arise independently in taxa exposed to comparable selective pressures (Anderson et al. 2024; Lankester 1870). The reliance on comparative methodologies underscores the necessity of a comparative framework, as it allows for meaningful comparisons between studies and species by addressing differences in terminology and methods (Pika et al. 2018).

Evidence from nonhuman species suggested that some features characterizing human conversational turn-taking may be widespread across the animal kingdom. Some features have been documented in non-primate mammals, including African elephants (*Loxodonta africana*; Soltis et al. 2005), beluga whales (*Delphinapterus leucas*) (Morisaka et al. 2013), and roe deer (*Capreolus capreolus*) (Reby et al. 1999); in birds, such as robins (*Erithacus rubecula*) (Dabelsteen et al. 1997), starlings (*Sturnus vulgaris*) (Henry et al. 2015), and large-billed crows (*Corvus macrorhynchos*) (Kondo et al. 2010); in anurans, including green frogs (*Rana clamitans*) (Wells 1977) and Sri Lankan tree frogs (*Philautus leucorhinus*) (Arak 1983); and in insects, such as North American katydids (*Amblycorypha parvipennis*), stoneflies (*Eucoptura xanthenes*), and tarbush grasshoppers (*Schistocerca obscura*; Bailey 2003) (Greenfield & Minckley 1993). These species all exhibit alternating vocal or auditory exchanges, the evolutionary basis of which remains unclear and may reflect shared evolutionary origins or convergent evolution (Pika et al. 2018). Although turn-taking is observed in many species, its structure is not uniform and may be species-specific, varying across multiple dimensions: form (e.g., flexibility of turn size), sequence (e.g., adjacency pair-like sequences), timing (e.g., inter-turn gaps), and distribution (e.g., turn allocation) (Pika et al. 2018; Sacks et al. 1974; Vlaeyen et al., submitted). In recent years, research interest in turn-taking has increased substantially, with researchers exploring vocal, gestural, and general turn-taking abilities in great apes (bonobos: Fröhlich et al. 2016a; Levréro et al. 2019; Rossano 2013; chimpanzees: Kolff & Pika, 2025; Pougnault et al. 2021; van Boekholt & Pika, 2025; western lowland gorillas, *Gorilla gorilla gorilla: *Pougnault et al. 2022). Concurrently, Afro-Eurasian monkeys have also been the subject of increased study. For instance, a study on adult Japanese macaques showed that, similarly to humans, individuals flexibly adjusted their response interval to the response latency of their interaction partners (Katsu et al. 2019). Likewise, studies on juvenile individuals of Japanese macaques and Campbell's monkeys (*Cercopithecus campbelli*) suggested that specific rules, such as call matching, needed to be learned to master turn-taking exchanges (Lemasson et al. 2011, 2013). Furthermore, red-capped mangabeys (*Cercocebus torquatus*) engaged in structured contact call exchanges, where overlapping calls were treated as violations, reflected by reduced listener attention (Meunier et al. 2024). These interaction patterns were further shaped by dominance rank and the level of social integration (Meunier et al. 2023). Based on existing evidence on turn-taking in great apes and monkeys, Levinson (2016b) proposed that vocal turn-taking may be ancestral within the primate order, with great apes having specialized in gestural rather than vocal turn-taking, a proposal hereafter referred to as the 'gestural specialization' hypothesis. Amid continuing debate about which modality most plausibly represents the evolutionary precursor of human language, identifying the modality underlying the emergence of turn-taking would therefore offer a significant contribution to this discussion (Arbib et al. 2008; Fröhlich et al. 2019; Pollick & De Waal 2007; Rodrigues et al. 2021). However, current knowledge of turn-taking in primates is limited by a research bias on several levels, including foci on experimental approaches, captive settings, vocalizations, specific features of conversational turn-taking, and particular species, especially common marmosets (see for an overview Pika et al. 2018). In monkeys specifically, gestural and facial modalities have received virtually no research attention. Consequently, there is not yet enough systematic quantitative data to verify the gestural specialization hypothesis, and clarify the evolutionary trajectory of turn-taking abilities and specializations in primates (Pika et al. 2018; Schegloff 2007; but see van Boekholt & Pika 2025).

In studies of primate turn-taking, grooming emerged as a key interactional context for examining interactional roles and how these roles are alternated and negotiated (Henzi & Barrett 1999; Pika 2014). Grooming serves a central function in maintaining social bonds and group cohesion, yet its performance is constrained by its high time and energy demands (Dunbar 1991). The "grooming-at-a-distance" hypothesis proposed that vocal communication evolved to maintain social bonds when direct grooming is impractical (Arlet et al. 2015; Dunbar 1991). This hypothesis is supported by observations that primates with stronger social bonds engaged in more frequent vocal exchanges, suggesting that vocalizations complement grooming to sustain social relationships across space (Levréro et al. 2019). This situates grooming within the evolutionary trajectory of vocal interactions, making it an ideal context to study the emergence of language-related abilities, such as turn-taking (Dunbar 1991; Kolff & Pika 2025).

In this study, we aimed to gain insight into turn-taking skills in an Afro-Eurasian monkey species, the sooty mangabey (*Cercocebus atys)*, living in their natural environment. Sooty mangabeys have been suggested to exchange calls, making them a useful candidate for studying turn-taking (Range & Fischer 2004). In addition, a closely related species, red-capped mangabeys, have been shown to communicate via gestures and employ them in intentional ways, with some similarities to great apes and other monkey species (Gupta & Sinha 2019; Molesti et al. 2020; Schel et al. 2022). Sooty mangabeys are a terrestrial member of the Cercopithecidae family and the Papionini tribe, which can be found along the west coast of Africa (Groves 1978, 2001). They live in multi-male and multi-female groups averaging 70 to 120 individuals, with females typically remaining in their natal group while males emigrate (Range & Noë, 2002). We specifically focused on social interactions of adult individuals since several studies have suggested that some elements of turn-taking systems may not be consistently exhibited in younger individuals (e.g., Abreu & Pika 2022; Lemasson et al. 2011; Takahashi et al. 2016). As in many primate species, grooming plays an important role in sooty mangabeys’ social life, functioning not only to maintain hygiene but also to reinforce social bonds within the group (Dunbar 2010). For instance, a study conducted at the Taï National Park, Côte d’Ivoire, showed that individuals dedicated approximately 15% of their activity budget to grooming (Fruteau et al. 2011). Grooming interactions lasted an average of five minutes and exhibited a high degree of within-interaction grooming reciprocity, with 97.8% of interactions involving bidirectional grooming (Fruteau et al. 2011). In addition, females typically focused their grooming efforts on two to four partners, who were often close in rank (Fruteau et al. 2011).

The present study addressed the following three key questions: (1) What turn-taking patterns, if any, characterize grooming interactions in sooty mangabeys? To address this question, we adopted the definition of Pika and colleagues (2018) (see also Vlaeyen et al., submitted), who view turn-taking as the structured and coordinated exchange of communicative signals or actions between two or more individuals, characterized by principles that regulate how turns are transferred and by temporal regularities. Signals and actions differ in that signals (e.g., facial expressions, gestures, or vocalizations) require a response from the recipient to achieve their intended outcome, whereas actions (e.g., grooming) can achieve their intended goal without eliciting a response from the recipient (Fröhlich et al. 2020; Genty et al. 2009; Hobaiter & Byrne 2011; Kolff 2025; van Boekholt 2024). We measured the presence of turn-taking by identifying the occurrence of turn transitions, which were defined as instances where one individual produced a unit (e.g., a vocalization, a gesture, a facial expression, or an action), and the next unit was initiated by the other individual (Sacks et al. 1974). Subsequently, to characterize the usage of turn-taking, we examined associations between the constituent units of turn transitions and quantified grooming reciprocity using a reciprocity index (Kolff 2025; Mitani 2009; Nishida 1988; van Boekholt et al. 2025). (2) Do sooty mangabeys preferentially use vocal turn-taking? In this study, we offered a first step to test the gestural specialization hypothesis (Levinson 2016b). To explore this question, we focused on vocalizations, facial expressions, and the production of gestures and tested whether sooty mangabeys engaged predominantly in facial turn-taking, gestural turn-taking, vocal turn-taking, or multimodal turn-taking (the first and second units of the turn transition are different modalities). (3) Which elements characterizing signal–signal turn-taking patterns of sooty mangabeys are shared with human conversational turn-taking? Given that different types of turn transitions may follow distinct coordination rules, we focused on the type that may be the most comparable to humans: signal-to-signal exchanges. For example, during turn-taking in pool skateboard sessions, the current rider did not select the next, the content of a turn bore no relation to preceding or subsequent turns, the end of a turn was not predictable, and riders avoided overlapping due to safety considerations (Ivarsson & Greiffenhagen 2015). These properties contrast with conversational turn-taking, where next speakers are allocated through a set of practices (including selection by the current speaker), turns are meaningfully connected to prior and subsequent turns, turn endings are often predictable at potential transition points, and overlap is handled through a more flexible set of coordination practices (Sacks et al. 1974). We examined three key features of human conversational turn-taking, following the framework of Pika et al. (2018):

- Element B, ‘who is taking the next turn,’ concerns the establishment of a participation framework (the initiator indicates who the intended recipient is and who will participate in the communicative exchange before producing a signal), and was assessed by investigating the parameters of gaze and body direction of the signaler during the production of signals used to initiate turn transitions and interactions.
- Element C, ‘when do response turns occur,’ refers to the temporal relationship between two turns. It was operationalized by calculating the intervals from the start of the initial unit to the start of the response, and from the end of the initial unit to the start of the response.
- Element D, ‘what should the next turn do,’ focuses on adjacency pairs and refers to two adjacent turns by participants, typically organized into first and second pair parts (e.g., question and answer, or a greeting and its response; Sacks et al. 1974; Schegloff 2007).

## Methods

### Study site and species

This study was conducted in the Taï National Park, Côte d’Ivoire, which spans 3,500 km2 and is the largest remaining area of primary forest in West Africa (Boesch & Boesch 1983). This dense evergreen forest is home to a diverse range of large mammals, including African forest elephants (*Loxodonta cyclotis*), African forest buffaloes (*Syncerus caffer nanus*), and leopards (*Panthera pardus*), as well as 10 different primate species (Boesch & Boesch 1983). Research has been ongoing in the park for decades, involving a long-term study on western chimpanzees (*Pan troglodytes verus*) since 1979 (Boesch & Boesch 1983).

For this study, we investigated the behavior of individuals of a group of sooty mangabeys, which consisted of around 70 individuals at the start of the first field season. The group has been habituated to human presence since 2013 (Mielke et al. 2017), allowing for the reliable identification of the majority of group members and the collection of high-quality behavioral and video data. In addition, for the majority of individuals in the group, extensive long-term behavioral and genealogical data were available.

### Data collection

Video data were collected by PT for a total of 12 months between May 2022 and August 2023 from 06:30 to 18:00. Continuous one-hour focal sampling was used on a total of 28 adult individuals (six males and 22 females) (Altmann 1974). Due to the small number of adult males and their frequent months-long absences during the study period, their data were not included in the subsequent analyses. Each focal individual was observed in a randomized order to prevent bias in the time of data collection. Following the completion of observations for all individuals, the sequence was reinitiated in a different randomized order. Particular attention was paid to ensuring systematic data collection across all subjects throughout each study month. One focal hour was counted if the focal individual had not been out of sight for more than 30 min in total, or 10 min consecutively. Using a digital camera (Sony Handycam FDR-AX100E) with an externally attached microphone (Sony ECM-CG60), recording of the focal animal took place at a distance of three to five meters when there was at least one other individual within five meters of the focal animal. Moreover, two trained research assistants collected direct observational behavioral data through half-day focal follows conducted from 06:30 to 18:00 (Altmann 1974). One observation day was counted when the group was followed for at least five hours.

### Data coding

Video recordings were extracted to a computer (Dell Latitude 5420) and coded using the program ELAN (version 6.4) (Hellwig and Sloetjes 2021). For nine non-related females, we coded 10 to 11 dyadic grooming interactions involving another female partner (Table 1). A maximum of one interaction per dyad was coded to capture a diverse range of social partners. Dyads were selected by prioritizing videos in which image quality, filming angle, and vegetation density provided optimal visibility of the behaviors performed during the interaction. Out of 22 adult females initially considered for data coding and analysis, the data of one female were excluded from the subsequent analysis due to her early disappearance during the data collection. This resulted in a total of 71 coded interactions, corresponding to approximately five hours of video footage. Of these, 27 interactions were complete, meaning that they captured both the beginning and the end of the grooming interaction. Behavioral definitions were based on behaviors described in ethograms of sooty mangabeys (Grampp et al. 2023; Range & Noë, 2002) and chimpanzees (Goodall 1986; Grund et al. 2024; Hobaiter & Byrne 2011; Pika 2014).

**Table 1:** Demographic and observational data of the individuals as a function of name (as a three-letter code), the approximate age of each subject (in years), total observation hours per individual (in hours), and the total number of coded grooming interactions per individual

Focals	Approximate age (years)	Observation hours	Coded interactions
SON	15	11	10
BZA	10	15	10
GIS	5	14	11
MER	7	13	10
AMB	17	13	10
TAS	7	16	10
LUS	9	14	10
RAM	9	16	10
MAL	6	14	10

^a^From here on, gestures are depicted in small capitals

For each interaction, the following information was coded:

- Identity of involved individuals.
- Interaction number: Unique number for the interaction.
- Actions performed by both individuals: Socially directed embodied behavior that leads to the perceived goal through direct manipulation of another’s body/body parts or the movement of one’s own body (Kolff & Pika 2025; van Boekholt & Pika 2025).
- Potential gestures and facial expressions: Socially produced, mechanically ineffective movement of the body, face, or limbs that could potentially result in a response from the recipient (Gupta & Sinha 2019; Pika 2008).
- Vocalizations: Vocalizations produced by both individuals during the interaction, based on the vocal repertoire of Range and Fischer (2004).
- Gaze and body direction of involved individuals (see Figure SM 1) (Abreu & Pika 2022; Pika et al. 2003; van Boekholt & Pika 2025; Vlaeyen et al., submitted):
  o Directed: angle from the midline (direction of the nose or nipples) and the body of the other individual ≤ 45 degrees
  o Not directed: angle from the midline (direction of the nose or nipples) and the body of the other individual > 45 degrees from the midline

Eighteen percent of the videos were coded by a second observer blind to the study’s hypothesis and research questions. A “substantial” to “almost perfect” agreement was obtained using ELAN’s Cohen’s Kappa coefficient from the “EasyDIAG” package using a 60% overlap (gestures: K = 0.95, vocalizations: K = 0.77, actions: K = 0.95, gaze direction: K = 0.96, body direction: K = 0.96) (Holle & Rein 2015).

### Behavioral operationalization & statistical analysis

#### Definition of grooming interactions and bouts

Following van Boekholt and Pika (2025), a grooming interaction was defined as an encounter between individuals that involved grooming. This began when one individual produced a signal or performed an action directed towards a recipient. The recipient could respond with a signal or action. The interaction ended when one individual moved away or when both individuals remained inactive for over 30 s (Aureli & Yates 2010; Goodall 1986; Kaburu & Newton-Fisher 2016; Newton-Fisher & Lee 2011). Grooming involved using one hand to move the partner’s hair aside, while the other hand inspected and cleaned the exposed skin, sometimes using the mouth to remove scabs, parasites, or foreign material (Goodall 1986). A grooming bout was defined as a distinct period of grooming engagement in which two individuals participated in continuous grooming, with one consistently acting as the groomer and the other as the recipient without switching roles (Foster et al. 2009). Therefore, a grooming interaction could consist of multiple grooming bouts if the roles of groomer and recipient changed during the interaction.

#### What turn-taking patterns, if any, characterize grooming interactions in sooty mangabeys?

To investigate the presence of turn-taking, we analyzed turn transitions. Turn transitions were classified into four categories based on the nature of the first and second units (signal or action): signal–signal, signal–action, action–signal, and action–action. Signal–action turn transitions present more basic forms of interaction, commonly observed across many species (Belding’s ground squirrel (*Urocitellus beldingi*), alarm whistle signal/flee-to-burrow response (Sherman 1977); jackdaw (*Corvus monedula*), allopreening solicitation posture/preening response (Katzir 1983)). By contrast, signal–signal turn transitions are used during conversational turn-taking and are seen in association with various conversation rules (e.g., overlap avoidance) (Sacks et al. 1974). Action–action turn transitions are also observed in humans, where they can share features of conversational turn-taking (Hofstetter 2021; Ivarsson & Greiffenhagen 2015; Sacks et al. 1974). For example, in board games, players manage turn transitions by monitoring when another’s move is nearing completion, recognizing natural endpoints, and using subtle cues (such as stepping back) to signal that the next player may begin (Hofstetter, 2021a). Interactions can include one type of turn transition or a combination of several with variable numbers and proportions.

To evaluate whether turn transitions occurred in a pattern consistent with random behaviors, we conducted a Chi-Square Goodness-of-Fit test. We first used the overall proportion of actions and signals across the dataset, which were approximately balanced at 52% and 48% respectively. Assuming that each turn occurred independently of the previous one, we calculated the expected frequencies for each transition type. These expected proportions were then converted into expected counts. We compared these expected counts to the observed transition counts using the Chi-Square test implemented using the chisq.test() function, with the rescale.p = TRUE option to match the scale of the observed data. We then conducted post hoc pairwise comparisons using pairwise proportion tests with Bonferroni correction using the function pairwise.prop.test(), to determine which transition types occurred significantly more or less often than others.

We used Multiple Distinctive Collocation Analysis (MDCA) with the program Coll.analysis 3.2a (Gries 2007), to measure the association between two units in a given turn transition. MDCA is a technique developed in linguistics to explore how often two specific words appear together (for review, see Gries 2013) and has recently also been applied to studies of animal communication (Bosshard et al. 2022; Kolff 2025). MDCA evaluates all possible unit combinations to see if they occur more or less frequently than chance would predict (Gries & Stefanowitsch 2004). Because it uses the binomial probability mass function, MDCA is particularly effective for analyzing skewed, non-random, and small datasets, which are often found in studies of animal communication (Bosshard et al. 2022; Gries & Stefanowitsch 2004; Leroux et al. 2021). The log-transformed results of MDCA (pbin) indicate the degree of exclusivity between combinations, with positive values showing a higher-than-expected occurrence and negative values indicating a lower-than-expected occurrence (Bosshard et al. 2022; Gries and Stefanowitsch 2004).

Within-interaction grooming reciprocity, that is, the alternation of grooming roles during a single interaction, has been documented in several primate species and represents a potential source of turn-taking (Chancellor & Isbell 2009; Fruteau et al. 2011; Newton-Fisher & Lee 2011). To assess whether it played a role in shaping turn-taking in sooty mangabeys, grooming reciprocity within interactions was quantified with a Grooming Reciprocity Index (GRI) (Mitani 2009; Nishida 1988). In this index, gAB represents the duration of grooming provided by individual A to individual B, and gBA refers to the grooming duration from individual B to individual A. The sum of gAB + gBA refers to the total grooming time between the two individuals. The index ranges from zero (indicating no reciprocity) to one (indicating complete reciprocity).

$$\text{GRI }=1- \left|\frac{{\mathrm{g}}_{\mathrm{AB}}}{{\mathrm{g}}_{\mathrm{AB}}+{\mathrm{g}}_{\mathrm{BA}}}-\frac{{\mathrm{g}}_{\mathrm{BA}}}{{\mathrm{g}}_{\mathrm{AB}}+{\mathrm{g}}_{\mathrm{BA}}}\right|$$

#### Do sooty mangabeys preferentially use vocal turn-taking?

Since gestures and facial expressions have not yet been studied in the context of grooming in sooty mangabeys, we first created a repertoire of the gestures and facial expressions produced by the focal animals in the observation period (but see Grampp et al. 2023). We defined gestures as movements of the extremities or head and body postures that can be directed to a social partner, are mechanically ineffective (produced to request or reach a goal, requiring active participation of the interactant), and potentially elicit a response by the recipient of the gesture (Pika 2008). Only the gestures and facial expressions that had been produced at least twice by two individuals or three times by one individual were included in the repertoire (Hobaiter & Byrne 2011; Pika et al. 2003). The repertoire was created by using the “verb first” principle (Nishida et al. 1999), as well as using, when possible, terms from previous gesture work on mangabeys and great apes (Aychet et al. 2021; Grampp et al. 2023; Pika et al. 2003, 2005; Schel et al. 2022). To ensure a comprehensive measurement of the entire gestural repertoire, we constructed a graph plotting repertoire size against the total number of observed gestures, aiming to reach an asymptote (Fröhlich et al. 2016b; Genty et al. 2009; Hobaiter & Byrne 2011). We assessed signal–signal turn transitions, which are cases where gestures, facial expressions, or vocalizations produced by one individual are directly followed by gestures, facial expressions, or vocalizations produced by another individual. They were categorized as facial, gestural, vocal, or multimodal turn transitions. To determine whether a dominant modality emerged in the distribution of turn transitions, we conducted a Chi-Square Goodness-of-Fit test comparing the observed counts of each category against an expected uniform distribution, where each category was assumed to occur with equal frequency (25%). We then performed pairwise comparisons using a **pairwise proportion test** with Bonferroni correction to identify which specific categories differed significantly from each other.

#### Which elements characterizing signal–signal turn-taking patterns of sooty mangabeys are shared with human conversational turn-taking?

We first investigated Element C by measuring the response time for each signal–signal turn transition. The onset-to-onset timing was measured as the time interval between the start of one unit by one individual and the start of the next unit produced by another individual (Kolff & Pika 2025; van Boekholt & Pika 2025; Vlaeyen et al., submitted). Although less commonly used (but see Fischer et al. 2000; Kolff & Pika 2025; van Boekholt & Pika 2025), it offers the advantage of circumventing methodological discrepancies related to gesture duration assessment (Grund et al. 2024). Offset-to-onset timing was measured as the interval between when the first unit produced by one individual ended and when the next unit produced by another individual began (Lemasson et al. 2013; Stivers et al. 2009; Takahashi et al. 2013; Vlaeyen et al., submitted). Concerning gestures, the offset was set when the gesture reached its final form, before its retraction, thus excluding the optional holding phase, which likely adds no semantic value (De Vos et al. 2015; Grund et al. 2024). When comparing signal–signal turn transitions to other types of turn transitions, the offset of actions was similarly defined as the moment when the movement required to achieve the intended goal ceased, excluding any subsequent retraction or holding movements (Vlaeyen et al., submitted). To determine whether a majority of turn transitions exhibited overlap avoidance, we performed a one-sided binomial test using the binom.test() function. We tested whether the observed number of turn transitions showing overlap avoidance was significantly greater than what would be expected by chance. The null hypothesis assumed that overlap avoidance occurred in half of the transitions, while the alternative hypothesis predicted a greater proportion, indicating a dominant tendency to avoid overlap. Additionally, we quantified the typical response window by identifying the time period during which the number of observed turn transitions exceeded the number of observed turn transitions for unlikely responses, defined as instances in which the second unit was potentially unrelated to the preceding unit (Lemasson et al. 2018; Levréro et al. 2019). This window thus reflects the timeframe in which responses are most likely to be genuine. Subsequently, we compared timings across turn transition types and communication modalities, enabling a deeper understanding of the factors shaping turn-taking timing. Differences were assessed using Kruskal–Wallis tests. Additionally, the overlap rate for each turn transition type and modality of signal–signal turn-taking was calculated. Differences across turn transition types were evaluated using Chi-squared tests, while differences across modalities of signal–signal turn-taking were assessed using Fisher’s exact test to account for the small sample size and ensure accurate inference.

Element B was assessed by measuring gaze and body direction parameters of signals used during signal–signal turn transitions. Within the 10 s preceding a signal being produced, we measured whether the gaze and body direction of the emitter were directed or not directed towards the recipient (Abreu & Pika 2022; Pika et al. 2003). We used one-tailed exact binomial tests to determine whether communicative behaviors were more likely directed toward the recipient (via gaze or body orientation) (50%). Three separate tests were conducted: one for gestures initiating signal–signal turn transitions, one for gestures initiating interactions, and one for vocalizations. No test was conducted for facial expressions, as none were observed in the species’ repertoire for the grooming context. Vocalizations initiating interactions were likewise not subjected to statistical analysis due to an insufficient number of observations, with only four instances recorded. To evaluate whether the proportion of signals accompanied by directed gaze and body orientation differed across signal types, we conducted proportion tests for overall comparisons. Post hoc pairwise comparisons using Fisher’s exact test were performed with Bonferroni correction to evaluate differences between the three signal categories: gestures initiating signal turn transitions, gestures initiating interactions, and vocalizations.

To evaluate Element D, we identified adjacency pair-like sequences. We used Multiple Distinctive Collocation Analysis (MDCA) to identify non-randomly associated signal pairs. To compare the timing of non-randomly associated signal pairs with all other events, we excluded the non-random pairs from the full dataset. We then performed a one-sided Wilcoxon rank-sum test to assess whether the offset-to-onset timings of non-random pairs were shorter (i.e., faster) than those of the remaining events.

All statistical analyses were performed using R version 4.3.2. Statistical significance was set at an alpha level of 0.05.

## Results

### Gestural repertoire

To allow further analyses, we compiled the repertoire of facial expressions and gestures for the focal individuals. We coded a total of 444 potential gestures and facial expressions, with 426 of them being subsequently classified into eight different gesture types (see Table 2). All gestures were either visual (generating a mainly visual component with no physical contact) or tactile (involving contact with the recipient) (Pika et al. 2003); no audible gestures (producing sound) or facial expressions met the criteria to be included in the repertoire. Based on the cumulative frequency of gesture types employed in the grooming context, it appeared that the group’s repertoire had reached an asymptote (Figure SM 2). This suggested that the full repertoire had been observed (Hobaiter & Byrne 2011; Pika 2007).

**Table 2:** Gestural repertoire of our study individuals in the context of grooming during the observation period, including the definition of each gesture, the number of occurrences, and the number of individuals observed performing the gestures ^a^(Hobaiter & Byrne 2011; Pika et al. 2003, 2005; Schel et al. 2022)

Gesture type	Definition^a^	Sensory modality	Number of occurrences	Number of individuals
embrace^a^	The signaler hugs the recipient by wrapping one or both arms or legs around him/her	Tactile	17	11
extend limb	The signaler stretches out a limb (arm or leg) horizontally toward or within the attentional field of the receiver	Visual	32	12
maintain contact	The signaler stops grooming, but one or both hands remain in touch with the body of the recipient for at least one second	Tactile	66	16
present	The signaler moves his/her body or body part to expose an area (excluding fingers, hand, toes, or foot) to the receiver’s attention and maintains that position	Visual	220	21
pull	The signaler’s hand or foot is firmly clasped over a part of the recipient’s body or a handful of hair and softly pulled toward the signaler	Tactile	18	10
push	The signaler makes contact with the recipient’s body, typically with a hand or a foot, exerting force	Tactile	13	10
raise	The signaler lifts a body part, such as a hand, arm, or leg, in a generally vertical movement, often pausing briefly near the top. A lifted hand involves the elbow not being above the shoulder; otherwise, it is considered lifting the arm	Visual	51	18
touch	The signaler lightly comes in contact with the recipient’s body, typically with fingers, knuckles, hand, or foot	Tactile	16	10

### Descriptive data

Direct behavioral observations allowed us to document a total of 595 grooming interactions among the 22 females. This dataset was used to assess the daily grooming rate.

For the video dataset, we conducted a total of 319.55 h of observation across 22 females (mean ± SD = 14.52 ± 1.55 h per female), yielding 105.3 h of video footage (mean ± SD = 4.79 ± 0.75 h per female). This resulted in 428 female–female grooming videos (mean ± SD = 19.45 ± 8.04 per female), of which 71 interactions were selected for coding. All other analyses were performed using this dataset.

Focal sooty mangabeys engaged in grooming an average of 2.21 times per day. Grooming interactions involved 1.34 ± 0.65 bouts on average (mean ± SD). The maximum of three bouts was observed in seven interactions. The 27 complete interactions lasted 95 ± 98 s on average (mean ± SD). In 90.77% of interactions with a recorded ending (59/65), the interaction ended with one individual moving away from the recipient by at least three meters. In the remaining cases, one individual was chased by a third individual, or both individuals stopped interacting for more than 30 s while remaining in close proximity.

In the coded videos, a total of 1,075 units were recorded, comprising 557 actions (mean ± SD = 57.15 ± 16.54% per dyad), 426 gestures (mean ± SD = 36.65 ± 13.93% per dyad), and 92 vocalizations (mean ± SD = 6.20 ± 12.09% per dyad). No facial expressions were observed (Tables SM 1 and SM 2, Figure SM 3).

### What turn-taking patterns, if any, characterize grooming interactions in sooty mangabeys?

A total of 92.96% of all observed interactions involved at least one turn transition, with an average of 7.86 ± 6.47 turn transitions per interaction (mean ± SD). In the remaining interactions, all behaviors were performed by a single individual, with the recipient passively receiving grooming and showing no further response. In total, 519 turn transitions were identified and analyzed. Of these, 55 transitions (10.40%) were classified as action–action, 165 (31.79%) as action–signal, 215 (41.61%) as signal–action, and 84 (16.19%) as signal–signal. Based on the overall behavior rates, the expected proportions were 27.04% for action–action, 24.96% for both action–signal and signal–action, and 23.04% for signal–signal. The observed distribution of turn transitions differed significantly from what would be expected under random usage (χ2 = 128.56, df = 3, p = 2.2 × 10⁻1⁶). This deviation indicated a structured, non-random pattern in how actions and signals are sequenced during turn transitions. Both mixed transitions, signal–action and action–signal, occurred significantly more often than action–action transitions (p = 9 × 10⁻3 and p < 2 × 10⁻1⁶, respectively). Signal–signal transitions were significantly less frequent than signal–action (p < 2 × 10⁻1⁶) and action–signal transitions (p = 3.6 × 10⁻⁸), but did not differ significantly from action–action transitions (p = 0.06). The average percentages of turn transitions per dyad were 13.12 ± 21.61% (mean ± SD) for action–action, 27.56 ± 18.34% (mean ± SD) for action–signal, 38.75 ± 22.49% (mean ± SD) for signal–action, and 13.53 ± 17.96% (mean ± SD) for signal–signal (Figure SM 4).

**Fig. 1:**
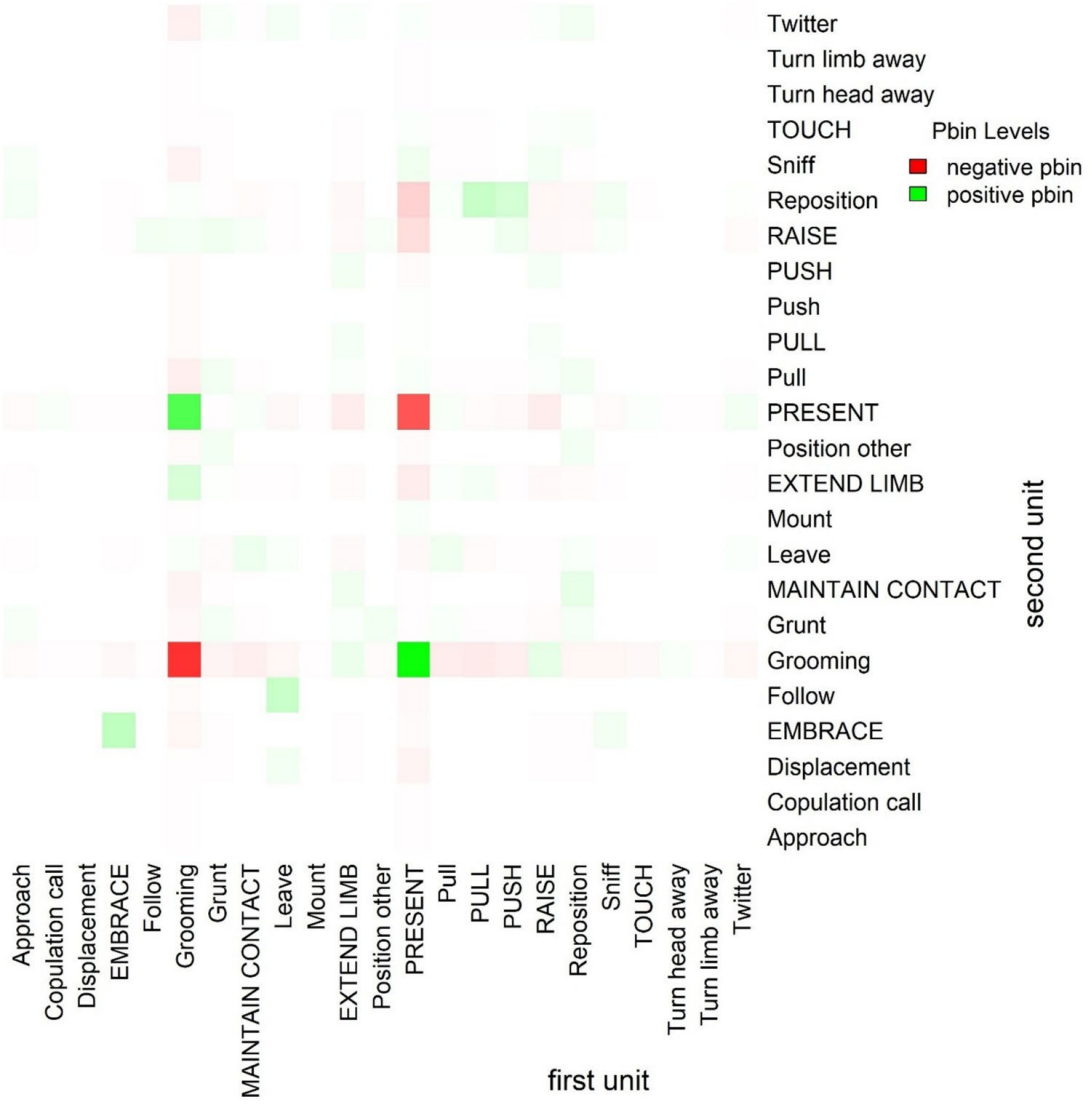
Matrix with columns and rows representing the first and second units within turn transitions, respectively. Positive pbin values in green represent higher-than-expected occurrences, negative values in red indicate lower-than-expected occurrences, and zero values appear in white. Color intensity reflects the magnitude of the pbin values, with more opaque colors indicating higher magnitudes (range: –22.43 to 27.03). Gestures are indicated in all capital letters

We found that 25 turn transitions were associated at a frequency higher than expected by chance. Among these, 15 were observed at least three times, thereby representing the most relevant pairs (Fig. 1 and Table SM 3) (Mielke & Carvalho 2022). For the remaining ten combinations, their relevance in the study individuals’ grooming interactions could not be fully established due to a relatively low production in the current dataset. The highest association was observed between the action grooming and the gesture present. Interestingly, grooming was strongly negatively associated with itself, similarly to present.

The average within-interaction grooming reciprocity index was 0.12 ± 0.25 (mean ± SD), indicating a low rate of reciprocity, and 23.94% of interactions included role reversals.

### Do sooty mangabeys preferentially use vocal turn-taking?

Signal–signal turn transitions were observed in 52.11% of dyads. Among these, 60.71% were gestural, 3.57% were vocal, 0% were facial, and 35.71% were multimodal (gesture and vocalization in either order) (Figure SM 5). We observed a highly significant difference from the expected equal distribution (χ2 = 83.143, df = 3, p < 2 × 10⁻1⁶). Gestural turn transitions were used significantly more than multimodal (p = 0.01), vocal turn transitions (p = 4.9 × 10⁻^14^), and facial turn transitions (p < 2.2 × 10⁻1⁶). Multimodal turn transitions were used more frequently than vocal turn transitions (p = 2.7 × 10⁻^6^) and facial turn transitions (p = 3.1 × 10⁻^8)^. No significant differences were found between vocal and facial turn transitions (p = 1.00).

### Which elements characterizing signal–signal turn-taking patterns of sooty mangabeys are shared with human conversational turn-taking?

**Fig. 2:**
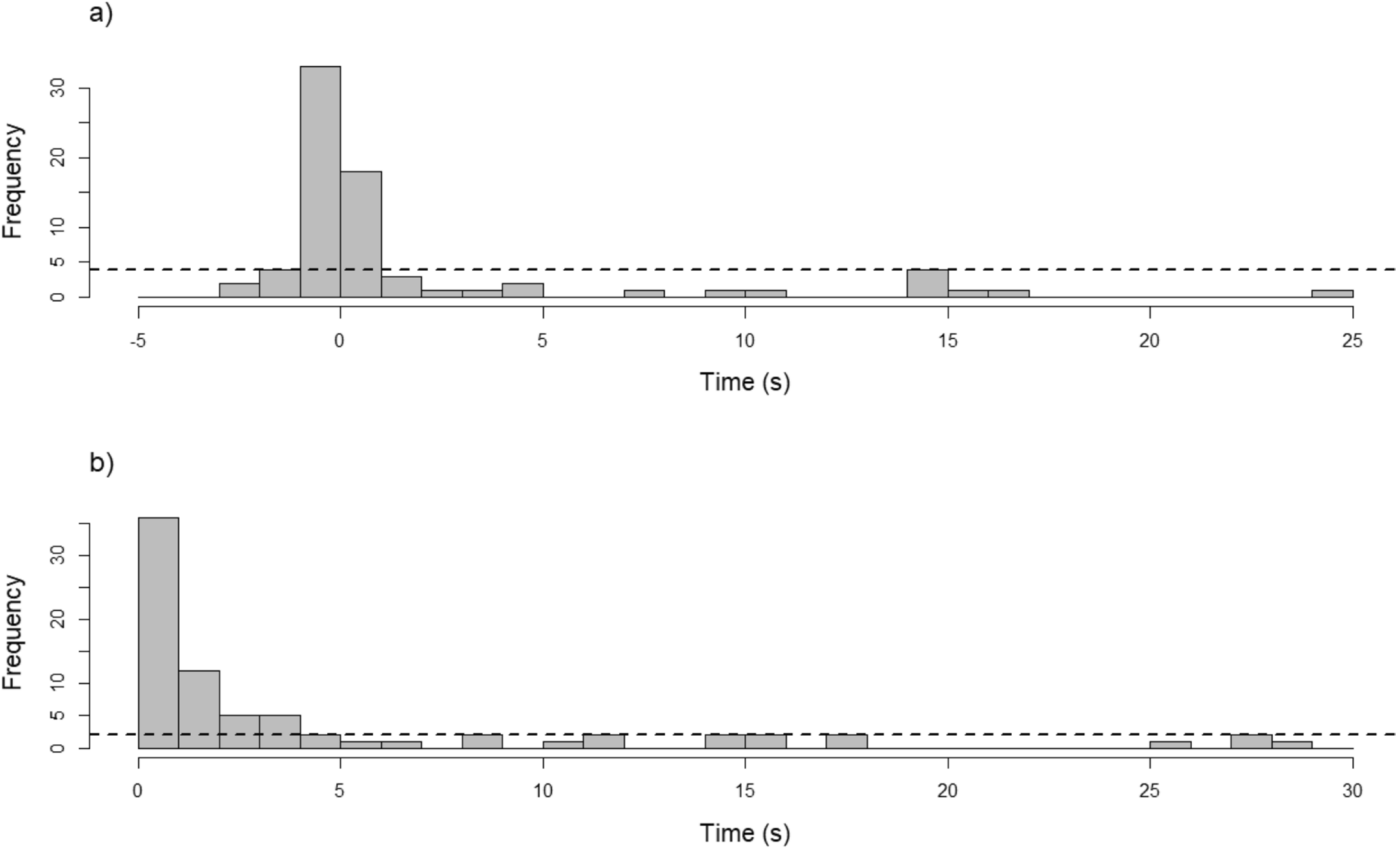
Overview of the temporal relationships of turns for signal–signal turn transitions with (a) showing the distribution of offset-to-onset gaps, and (b) depicting the distribution of onset-to-onset gaps. To improve readability, the plot was limited to the range from − 5 s to 25 s for (a) and 0 s to 30 s for (b)

Examining element C, ‘when do response turns occur,’ we found that onset-to-onset gaps of signal–signal turn transitions were highly variable, ranging from 0.009 s to 109.85 s (mean ± SD = 9.16 ± 18.86 s, median = 1.52 s) (Fig. 2). Similarly, offset-to-onset gaps spanned from − 2.97 s to 109.61 s (mean ± SD = 7.72 ± 19.09 s, median = 0.09 s) (Fig. 2). Negative offset-to-onset gaps, representing overlapping turn transitions, occurred in 41.67% of cases, which was not significantly different from 50% (p = 0.156), suggesting that overlap avoidance did not dominate in this dataset. To identify the typical response times, we analyzed histograms of turn transitions. A horizontal threshold line was drawn that represents how often unlikely response turn transitions or uncommon response intervals occur (Lemasson et al. 2018; Levréro et al. 2019). Intervals above this line fell inside the typical range. Based on this criterion, responses generally occurred within 1 s of the offset of another signal or within 4 s of the onset of a signal. The typical median onset-to-onset was 0.68 s (mean ± SD = 1.08 ± 0.99 s) and the typical median offset-to-onset was − 0.09 s (mean ± SD =  − 0.32 ± 0.75 s). Within the range of typical responses, 61.40% of offset-to-onset turn transitions overlapped, with a mean overlap duration of 0.71 s.

Signal–signal turn-taking exhibited shorter onset-to-onset durations compared to other transition types (all transitions: median = 2.89 s; action–action: median = 8.32 s; action–signal: median = 14.51 s; signal–action: median = 1.59 s) (Figure SM 6). The difference between the timing of these turn transitions was significant (χ^2^ = 131.93, df = 3, p < 2 × 10⁻1⁶). Signal–signal turn-taking exhibited shorter offset-to-onset durations compared to other transition types (all transitions: median = 0.35 s; action–action: median = 1.18 s; action–signal: median = 0.55 s; signal–action: median = 0.13 s) (Figure SM 7). However, these differences were not statistically significant (χ^2^ = 6.277, df = 3, p = 0.099). The rate of overlap was higher for signal–signal transitions (41.67%) compared to action–action (29.63%) and signal–action (37.5%), and similar to action–signal (41.21%) transitions, but these differences were likewise not statistically significant (χ^2^ = 1.250, df = 3, p = 0.741).

Gestural offset-to-onset turn transitions had a mean duration of 5.82 s and a median of 0.09 s, with an overlap rate of 45.1%. Multimodal turn transitions were longer, with a mean of 11.69 s and a median of 0 s, and an overlap rate of 33.3%. Vocal turn transitions were shorter, with a mean of 0.47 s and a median of 0 s, but exhibited the highest overlap rate at 66.7%. However, the differences in timing across modalities were not significant (χ2 = 3.040, df = 2, p = 0.219), nor were the differences in overlap rates (p = 0.405).

For element B, ‘who is taking the next turn,’ directed gaze accompanied gestures that initiated signal–signal turn transitions in 56.45% of cases (35/62, p = 0.1871), which did not differ significantly from chance. Directed body orientation occurred in 67.74% of gestures that initiated signal–signal turn transitions (42/62, p = 3.57 × 10⁻3), a proportion significantly above chance. Focusing specifically on gestures that initiated interactions, directed gaze was observed in 79.32% of cases (23/29, p = 1.16 × 10⁻3), and directed body orientation in 75.86% (22/29, p = 4.07 × 10⁻3), both significantly above chance. For vocalizations that initiated signal–signal turn transitions, directed gaze accompanied the vocalization in 88.24% of cases (15/17, p = 1.18 × 10⁻3), and directed body orientation occurred in 76.47% (13/17, p = 2.45 × 10⁻2), both exceeding chance levels. Comparing directed gaze across these signal types revealed a significant overall difference (p = 1.33 × 10⁻^2^). Post hoc pairwise comparisons did not reach significance (signal–signal gestures vs. initiating interaction gestures, p = 0.12; signal–signal gestures vs. vocalizations, p = 6.54 × 10⁻2; initiating interaction gestures vs. vocalizations, p = 1.00), likely due to reduced statistical power from splitting the data and applying Bonferroni correction. For directed body orientation, differences across these signal types were not significant (p = 0.64), indicating similar orientation rates across categories.

For element D, ‘what should the next turn do,’ we found that three signal pairs were not randomly associated (push—raise, grunt—raise, embrace—embrace). The 11 non-randomly associated turn transitions had a median offset-to-onset timing of − 0.24 s, compared with 0.09 s for all other events excluding these non-random pairs, a difference that was statistically significant, indicating that non-random pairs occurred faster (p = 0.035). Notably, these pairs exhibited a high overlap rate of 81.82%.

## Discussion

This study aimed to contribute to testing the hypothesis that turn-taking constitutes an ancient layer of human language, conserved across all major primate clades, reflecting phylogenetic continuity (Levinson 2016b; Pika et al. 2018). To address this objective, we investigated turn-taking interactions in female sooty mangabeys living in their natural environment with a special focus on the cooperative context of grooming. We examined three key questions: (1) What turn-taking patterns, if any, characterize grooming interactions in sooty mangabeys? (2) Do sooty mangabeys preferentially use vocal turn-taking? and (3) Which elements characterizing turn-taking patterns of sooty mangabeys are shared with human conversational turn-taking?

### What turn-taking patterns, if any, characterize grooming interactions in sooty mangabeys?

We found that nearly all grooming interactions in sooty mangabeys involved turn-taking. These findings mirror recent results of a study on turn-taking in male Eastern chimpanzees (*Pan troglodytes schweinfurthii*) showing that more than eighty percent of all grooming interactions included at least one turn transition (Kolff 2025). These findings suggest that turn-taking could be a widespread feature in grooming interactions among these two species. This behavior helps to mediate grooming interactions either to enable even distribution of benefits and balance the roles of grooming and being groomed, to negotiate change in grooming location on the body, or both.

Like chimpanzees, the interactions of the sooty mangabeys studied here also combined all types of turn transitions (Kolff 2025; van Boekholt & Pika 2025). This interactional pattern could represent a transitional stage toward signal-based conversational turn-taking, a feature considered foundational in the evolution of language (Levinson 2016b). Nevertheless, differences between chimpanzees and sooty mangabeys were evident. We found that action–signal and signal–action transitions dominated sooty mangabeys’ grooming interactions, whereas in adult male chimpanzees, action–action and signal–action transitions were most frequent, with signal–signal occurring at comparable rates in both species (Kolff 2025). Turn-taking patterns in mother-infant chimpanzees differed further, with all types of turn transitions used at balanced rates during food-sharing, grooming, joint travel, and nursing (van Boekholt & Pika 2025). Thus, sooty mangabeys predominantly relied on signal–action turn transitions, whereas adult chimpanzees more frequently employed action–action turn transitions, which in humans share some, but not all, organizational properties with conversational turn-taking (Hofstetter 2021; Ivarsson & Greiffenhagen 2015). One possible explanation for these differences is that, unlike in chimpanzees, grooming by individual A in mangabeys was rarely immediately followed by grooming from individual B (action–action) (Kolff 2025). Role reversals were relatively rare overall, and when they occurred, they were often facilitated by communicative signals (action–signal followed by signal–action). These patterns may reflect species-specific social dynamics and communication styles: chimpanzees more frequently engage in mutual and reciprocal grooming, which requires little signaling, whereas sooty mangabeys rely more heavily on communicative signals to coordinate the less common grooming role reversals (Fröhlich et al. 2016a). Another contributing factor may be that sooty mangabeys have a larger number of grooming partners relative to chimpanzees (Newton-Fisher & Lee 2011; Watts 2000). This may lead to lower familiarity with each partner and necessitate more explicit communicative signals to coordinate grooming, such as indicating when to change grooming location. This interpretation is supported by the frequent use of gestures aimed at presenting a specific body part for grooming, including present, extend limb, and raise. However, this hypothesis remains tentative, as evidence from male Eastern chimpanzees suggests that the social bond strength does not significantly influence the type of turn transition employed during grooming (Kolff & Pika 2025). Overall, this cross-species comparison suggests that action–action turn transitions in grooming interactions may be favored in groups where reciprocal grooming is common and expectations are predictable. By contrast, lower partner familiarity, and infrequent role reversals appear to increase reliance on communicative signals, thereby enhancing the prevalence of action–signal exchanges. However, since the present study is based on a single sooty mangabey group, further work is needed to determine how broadly these findings apply across the species. Additionally, direct cross-species comparisons using standardized methods are necessary to clarify how social and ecological factors shape patterns of turn transition use during grooming coordination and across other interactional contexts. This will allow for the identification of the mechanisms that facilitate signal–signal turn transitions and conversation-like interactions, and clarify their role in the evolutionary origins of conversational turn-taking and language.

This study revealed that two action–signal pairs, grooming-present and present-grooming were the predominant pairs observed. This pattern indicates that communicative exchanges during grooming interactions in our study individuals were predominantly focused on the location of grooming (“groom here”) rather than on the negotiation of grooming roles (“you groom me”) and grooming tasks (“your turn now”). A total of 15 combinations were frequently associated at a rate higher than predicted by chance. Some were variations of present-grooming, such as raise-grooming and extend limb-grooming, while others involved changes in body posture, including pull-reposition and push-raise.

Similarly, in the study on adult Eastern chimpanzees, nine non-randomly associated signal–action pairs were observed during grooming, four of which were identical to those found in sooty mangabey grooming interactions: present-grooming, raise-grooming, pull-reposition, and push-reposition (Kolff 2025). These similarities suggest that in both species, in grooming interactions, turn-taking is used to facilitate coordinated exchanges, specifically, requests for grooming and changes in the recipient’s body position. Further data and comparative studies are needed to clarify the role of exchanges produced during turn-taking in grooming interactions and beyond, providing insight into the contexts in which turn-taking occurs, the types of interactions in which it first emerged, and how it may have evolved.

### Do sooty mangabeys preferentially use vocal turn-taking?

Concerning question two, we found that our study individuals used two different modalities during signal–signal turn-taking: gestural, and vocal. They combined them in multimodal turn transitions. Although the female mangabeys showed some instances of vocal turn-taking using grunt and twitter calls, only gestural adjacency pair-like sequences were observed. Whereas vocal turn-taking has been suggested to exist in sooty mangabeys (Range & Fischer 2004) and other Afro-Eurasian monkey species (Katsu et al. 2019; Lemasson et al. 2011; Richman 1976), investigations into gestural signaling were, until now, very limited (but see, Gupta & Sinha 2019; Laidre 2011; Molesti et al. 2020). Our study reveals that, in this specific context of grooming, gestural and multimodal signaling were the most crucial means, emphasizing the need to gain a more detailed understanding of non-vocal communicative modalities beyond the great ape species. Our results also challenge the hypothesis that great apes are the taxa specialized in gestural rather than vocal turn-taking, suggesting instead that gestural turn-taking may represent an early-emerging feature of turn-taking across the primate lineage (Levinson 2016b). Amid persistent debate over the modality in which language originated, this study provides support for the view that gestural communication played a crucial role in the emergence of language-linked cognitive and communicative capacities (Arbib et al. 2008; Fröhlich et al. 2019; Pollick & De Waal 2007; Rodrigues et al. 2021). In chimpanzees, grooming and play contexts preferentially involved gestural signals, whereas feeding interactions were dominated by vocalizations (Fröhlich et al. 2016c, 2020; Hobaiter et al. 2017). Modality choice was shaped by factors such as individual matrices, mechanical constraints, social circumstance, the potential cost of misinterpretation, and the degree of privacy afforded by different signal types (Fröhlich et al. 2016c, 2017; Hobaiter et al. 2017). These findings highlight the importance of examining multiple interactional contexts to characterize broader patterns of modality use for turn-taking within a species. Accordingly, systematic comparative studies examining the frequency and complexity of gestural and vocal turn-taking across apes and other primate species in various contexts are needed to fully evaluate this hypothesis, and such data are currently lacking (but see Southern et al., submitted).

### Which elements characterizing signal–signal turn-taking patterns of sooty mangabeys are shared with human conversational turn-taking?

Regarding question three, we examined three hallmark features of human conversational turn-taking with the aim of comparing them across primate taxa. This comparison aimed to identify shared characteristics and to test the interaction engine hypothesis, which proposes that these features emerged through homology within the primate lineage.

With regard to element C, ‘when do response turns occur,’ we found that despite the wide range of response times, the studied sooty mangabeys interacted within a relatively narrow window of a few seconds. Such specific timing likely plays a critical role in enabling both the signaler and the recipient to recognize that they are engaged in a coordinated interaction. This temporal framework may thus aid in clearly delineating the boundaries, beginnings, and endings of turns, thereby facilitating smoother coordination during grooming.

Compared to humans, the typical median signal–signal offset-to-onset found in this study (− 0.09 s) was faster than the timing reported in a study investigating conversational turn-taking across several different languages (0.10 s) (Stivers et al. 2009). Turn-taking timing in sooty mangabeys was more variable, evidenced by a comparatively higher standard deviation in typical offset-to-onset timing (Stivers et al. 2009). Turn-taking during grooming interactions did not involve overlap avoidance, contrary to conversational turn-taking (Sacks et al. 1974). The overlap rate of typical answers was one and a half to four times higher than that observed in human question–answer interactions across spoken languages and sign languages (Beukeleers & Lepeut 2023; De Vos et al. 2015; Heldner 2011). However, it is important to note that turn-taking timing in humans can be influenced by factors such as age and social relationships (Beaumont & Cheyne 1998; Dewi & Munir 2018; Sprecher & Treger 2015). Investigating these variables in future research may enable more meaningful comparisons and deepen our understanding of the evolution of turn-taking dynamics.

To date, no turn-taking studies have been conducted on sooty mangabeys. In red-capped mangabeys, typical response times for the exchange of contact calls fell within 2 s, although overlapping exchanges were rare, in contrast to what we observed (Meunier et al. 2023). The reported average response time of 0.5 s is slower than our observed typical average of − 0.32 s, although this difference may result from the study’s exclusion of overlapping calls (Meunier et al. 2023). Compared to other studies on Afro-Eurasian monkeys, the typical signal–signal offset-to-onset timing observed in this study was faster than that reported for vocal turn-taking in Japanese macaques and Campbell’s monkeys. The authors reported that overlapping calls were extremely rare (Katsu et al. 2019; Lemasson et al. 2010). This difference may be due to inherent distinctions between communication modalities. It has been proposed that vocal communication involves less overlap than visual gestural communication because overlapping vocalizations can lead to perceptual masking and production difficulties, while overlapping visual gestures are typically easier to perceive simultaneously without such interferences (Coates & Sutton-Spence 2001; Emmorey et al. 2009). Some gestures observed in this study, such as pull or touch, included a tactile component. Research has shown that the somatosensory system has a limited capacity to process multiple tactile inputs simultaneously (Kusnir et al. 2023). Even so, issues with overlap between turns would likely arise only when individuals are both receiving and producing tactile gestures without visual contact, an uncommon scenario, as it would be difficult to accurately touch someone without seeing them. Our findings did not provide evidence that gestural turn transitions permit greater overlap than vocal turn transitions. However, the low frequency of vocal turn transitions constrained the interpretability of these comparisons. Further studies in Afro-Eurasian monkeys are needed to determine whether communicative modality influences turn-taking timing. Additionally, the present study did not exclude the preparation phase of gestures from the analysis. It has been argued that these phases should be omitted (McCleary & Leite 2024) because, in vocal communication, pre-vocal preparations such as in-breaths and pre-speech tongue movements are usually not included in response-time measures (Palo et al. 2014). Excluding gestural preparation phases might therefore have produced response times more comparable to those reported for vocal exchanges.

When contrasted with results from studies on great apes, the general median offset-to-onset timing in sooty mangabeys (0.35 s) was faster than that observed in joint travel between mother-infant chimpanzees and bonobos, which was close to 1 s (Fröhlich et al. 2016a). However, it was closer to that reported for adult male Eastern chimpanzees during grooming interactions, who exhibited no delay between turns (Kolff & Pika 2025). These differences suggest that developmental factors may influence the timing of turn-taking in chimpanzees (Fröhlich et al. 2016a; Lemasson et al. 2013). Similarly to chimpanzees, overlap avoidance, a key feature of human conversational turn-taking, was not observed during grooming interactions in sooty mangabeys (Kolff 2025). This suggests that sooty mangabeys anticipated the goal of the signaler and initiated their response before the signal had ended (Badihi et al. 2024; Kolff 2025). Despite slight differences, the offset-to-onset timings of turn-taking observed in our study were rapid, similar to those observed in other Afro-Eurasian monkeys, great apes, and humans, further contributing to the growing body of evidence supporting ancestral quick turn-taking abilities in the primate lineage (Abreu & Pika 2022; Levinson 2016b; Pika et al. 2018).

Onset-to-onset timing is typically not presented in studies on humans or Afro-Eurasian monkeys. Relative to great apes, the observed general median onset-to-onset timing (2.89 s) was longer than that reported for mother-infant and adult chimpanzees, whose median timings were closer to two seconds (Kolff 2025; van Boekholt & Pika 2025). This difference may be attributed to the nature of the grooming context, where units are structured to be relatively long, while the study on mother-infant chimpanzees also focuses on the contexts of food-sharing, joint travel, and nursing (van Boekholt & Pika 2025). Another explanation could be that due to their bigger neocortex and involved neurons, chimpanzees may require less pre-processing time than sooty mangabeys, enabling them to initiate responses quicker (Herculano-Houzel 2017). This interpretation supports the broader hypothesis that chimpanzees possess higher cognitive processing abilities than Afro-Eurasian monkeys (Byrne 1995; Matsuzawa 2010).

Regarding element B, ‘who is taking the next turn,’ we found that gaze and body direction were used to initiate interactions and during signal–signal turn transitions, suggesting that these cues play a role in coordinating interactions. Specifically, we observed that directed body orientation was significantly present in signal–signal initiating gestures, interaction-initiating gestures, and vocalizations, whereas directed gaze was significantly present only for interaction-initiating gestures and vocalizations. The role of directed eye gaze during vocal turn-taking remains largely unexplored. Our study provided evidence that vocalizations used during social interactions were frequently accompanied by a directed gaze toward the recipient. Research on chimpanzees has shown that in the alarm context, they similarly use directed gaze specifically during certain vocalizations, such as Alarm Hoots and Waa Barks, although this behavior has not been documented during turn-taking interactions (Schel et al. 2013).

When comparing with human communication, we observed that in both species, directional cues play a role in managing turn-taking. Human language is hypothesized to have evolved primarily for face-to-face interactions, with gaze playing a central role (Kendon 1967; Levinson 2016b). Eye gaze is not only used to signal the end of a turn but also to manage repair and interruptions (Brône et al. 2017; Ho et al. 2015; Kendon 1967; Rossano, 2009, 2013; for review, see Degutyte & Astell 2021). However, the use of directed gaze varies depending on the type of interaction. For instance, questions are more likely to be accompanied by a directed gaze toward the recipient, whereas free-flowing conversation or storytelling often involves less eye contact (Eberhard & Nicholson 2010; Kendon 1967; Rossano et al. 2009). Cultural differences further influence these dynamics, with studies reporting that in some cultures, questions are less likely to be asked while looking at the recipient than in others (Rossano et al. 2009). Research on gaze direction duration during free-flowing conversations remains limited; nevertheless, one study found that Japanese speakers maintained eye contact with their conversational partner only around a quarter of the time while speaking (Ijuin et al. 2018). Further studies could investigate whether, as in humans, contextual and social factors influence gaze behavior during turn-taking in sooty mangabeys. Such research may enable holistic comparisons and contribute to our understanding of the evolution of participation framework establishment in turn-taking.

Concerning findings on other Afro-Eurasian monkeys, our results showed that just over half of the gestures used to initiate signal–signal turn transitions involved directed gaze. This contrasts with previous studies reporting that about ninety percent of gestures in captive olive baboons and nearly all gestures observed in captive red-capped mangabeys were produced with directed gaze toward the recipient (Molesti et al. 2020; Schel et al. 2022). Differences between the studies may be due to the use of different methodologies, with the two above-mentioned studies focusing on other contexts, such as affiliation, aggression, sex, and social play, rather than only the grooming context. In a context where both interactants are in body contact and where body positions are constrained, such as grooming, checking the attentional state of the recipient before signaling could be less common than in other contexts. This finding should be considered in future research examining gaze and body direction in the grooming context (for example, for first-order intentionality). Furthermore, we argue that gaze may play a different role with regard to the function and intended meaning of the accompanying signal. For instance, if an individual “opens” the interaction and “conversation” (Pillet-Shore 2018), then directed signals may be crucial to emphasize this request and the transferred meaning. In contrast, in an ongoing and already established interaction, directed signals might not be needed to transfer the information and reach the desired goal. This hypothesis is supported by our data, which showed that direct eye gaze was employed primarily at the initiation of interactions rather than continuously throughout.

In great ape studies, some researchers classify a behavior as a gesture only if it includes markers of intentional signalling such as goal persistence and directed gaze (Cartmill & Byrne 2010; Genty et al. 2009; Hobaiter & Byrne 2011; Pollick & De Waal 2007). This approach excludes gestures that occur within an already established interaction and no longer require directed gaze (e.g., tactile gestures used to request a change in the recipient’s position), or that are immediately successful, such that response waiting or goal persistence is no longer necessary. However, some studies differentiated between intentionally produced gestures showing more than one key characteristic of intentional communication and gestures showing only one key characteristic of intentionality (Fröhlich et al. 2016a), enabling a more comprehensive understanding of gestural production and usage. They also showed that in mother-infant dyads of chimpanzees and bonobos, signalers used directed eye gaze and body orientation to initiate joint travel in fewer than sixty percent of cases, a higher rate than that observed in sooty mangabeys (Fröhlich et al. 2016a). Notably, species- and age-related differences were apparent: bonobos relied more on directed eye gaze than chimpanzees, while older infants of both species tended to use body orientation more frequently than younger ones (Fröhlich et al. 2016a). These findings suggested that gaze direction during turn-taking may be subject to developmental and inter-species variations, which may help explain the discrepancies between our findings and those reported in this previous study. Despite varying rates, great apes and the sooty mangabeys of the present study used directional cues during turn-taking, suggesting that this element of the conversational turn-taking system may have ancient evolutionary roots across the primate lineage.

The analysis of element D, ‘what should the next turn do,’ identified three signal sequences akin to adjacency pair-like sequences, which were both more frequently associated than expected by chance and faster than other types of turn transitions.

When compared to humans, our study revealed that grooming interactions in sooty mangabeys were similarly organized into signal adjacency pair-like sequences, which occurred faster than other turn transitions (Roberts et al. 2015; Sacks et al. 1974). This indicated that signal exchanges were not random or incidental, but instead formed a characteristic feature of mangabeys’ grooming interactions. We also found that adjacency pair-like sequences showed a high rate of overlap, which stands in contrast to the general avoidance of overlap documented for human conversation (Sacks et al. 1974). This pattern may reflect the nature of the specific pairs involved. The push–raise pair (e.g., individual A gently pushing individual B’s arm, followed by individual B lifting that arm) resembles agreement sequences, which are known to display increased overlap (Pomerantz 1984; Vatanen 2018). The embrace–embrace pair (i.e., mutual hugging) is comparable to reciprocal greeting sequences, where overlap is also reported (Pillet-Shore 2012). Because embrace involves tactile contact, additional mechanical constraints may further contribute to overlap. Further work will be useful to determine whether adjacency pair-like sequences in sooty mangabeys and other primate species reliably exhibit elevated overlap and to identify the mechanisms that produce this pattern. In this study, we did not assess the intentionality or communicative meaning of the signals involved, which would strengthen the claim that the second unit functioned as a contingent response to the first (Schegloff 2007). Addressing this limitation should be a key objective for future research seeking to identify and analyze structured patterns of communicative exchange.

Considering Afro-Eurasian monkeys, studies demonstrating adjacency pair-like sequences remain rare. However, Lemasson and colleagues (2011) highlighted call matching in Japanese macaques, suggesting a conversational rule in which vocalizations were answered with matching calls. Although this indicated that vocal interactions in Japanese macaques were governed by structured rules, similar evidence for other modalities and species remains lacking. Regarding great apes, our results were consistent with observations of adult Eastern chimpanzees and mother-infant chimpanzees, where adjacency pair-like sequences have also been documented (Fröhlich et al. 2016a, b; Kolff 2025; van Boekholt et al. 2025). However, since some studies focused exclusively on gesture–response pairs, direct comparisons of the modalities and units used are not possible (Fröhlich et al. 2016a, b; Kolff 2025). Our results contrast with findings on interactions of mother-infant chimpanzees, where signal–signal adjacency pair-like sequences have not been observed; instead, only either signal–action (e.g., open arm–nursing) or action–action pairs (e.g., nursing–grooming) have been described (van Boekholt et al. 2025). Further research, particularly involving adult individuals, is necessary to determine whether such patterns emerge at later developmental stages, as age has been shown to influence the production and use of gestures, and gesture-response pairs (Fröhlich et al. 2016a; Pika 2007). 

To date, mother-infant ape interactions have served as the primary model for investigating gestural turn-taking (Fröhlich et al. 2016a; Rossano 2013). However, our findings suggested that systematic studies into the behaviour of Afro-Eurasian monkeys living in their natural environments may offer valuable insights and could serve as promising subjects for advancing research in this domain.

Taken together, the present findings add another piece of evidence to support the hypothesis that turn-taking is an ancient mechanism already present across the whole primate lineage (Levinson 2016b, 2019; Levinson & Holler 2014; Pika et al. 2018). More specifically, these abilities may have already been present in the last common ancestor of humans and catarrhine monkeys, which lived approximately 20 to 30 million years ago (Raaum et al. 2005; Steiper & Young 2006). This is consistent with the idea that turn-taking constitutes one of the earliest layers of human communication.

## Conclusion

This study explored turn-taking patterns in grooming interactions among adult female sooty mangabeys living in a single wild population. The findings strengthened the view that turn-taking is a fundamental feature of primates' social and cooperative exchanges. Importantly, this research provided evidence of gestural turn-taking in Afro-Eurasian monkeys, underscoring the importance of expanding comparative studies to the wide diversity of monkey species. Future studies will be important to test the gestural specialization hypothesis (Levinson 2016b). Comparisons with temporal features and the organization of interactions into adjacency pair-like sequences revealed parallels with humans and great apes. Similarly, the use of directional cues aligned with communication patterns in both humans and non-human primates. These similarities suggested that the roots of conversational turn-taking could extend deep into primate evolution. Continued comparative research will be essential to unravel how the building blocks of language have evolved in the primate lineage.

## Supplementary Information

Below is the link to the electronic supplementary material.

Supplementary file1 (DOCX 225 KB)

Supplementary file2 (PDF 53 KB)

Supplementary file3 (PDF 8 KB)

Supplementary file4 (PDF 64 KB)

## Data Availability

The data generated by this study are available at 10.6084/m9.figshare.29304983.
