# Supplementary material for: Turn-taking in grooming interactions of sooty mangabeys (Cercocebus atys) in the wild
Source: Anim Cogn. 2026 Mar 10;29(1):40. doi: 10.1007/s10071-025-02040-2 (PMC13102903; doi:10.1007/s10071-025-02040-2)
Supplement: Supplementary file 1 — Supplementary file1 (DOCX 225 KB) [file 10071_2025_2040_MOESM1_ESM.docx]

Turn-Taking in Grooming Interactions of Wild Sooty Mangabeys (*Cercocebus atys*)

Animal cognition

**Pharisemène Tibesar^1^, Catherine Crockford^234^, Auriane Le Floch^2356^, Simone Pika^1^**

^1^Comparative BioCognition, Institute of Cognitive Science, Osnabrück University, Artilleriestrasse 34, 49076, Osnabrück, Germany

^2^ The Ape Social Mind Lab, Institut des Sciences Cognitives Marc Jeannerod, CNRS, 67 Boulevard Pinel, 69675 Bron, Lyon, France

^3^Taï Chimpanzee Project, Centre Suisse de Recherches Scientifique en Côte d’Ivoire, Abidjan, Ivory Coast

^4^Department of Human Behaviour, Ecology and Culture, Max Planck Institute for Evolutionary Anthropology, 04103 Leipzig, Germany

^5^ Institute of Biology, University of Neuchâtel, Neuchâtel, Switzerland

^6^Taï Monkey Project, Centre Suisse de Recherches Scientifique en Côte d’Ivoire, Abidjan, Ivory Coast

**Corresponding author:**

Pharisemène Tibesar

ftibesar@uni-osnabrueck.de

**Table SM 1** Coded actions along with their definitions. ^a^(Goodall, 1986; Hobaiter & Byrne, 2014; Nishida et al., 1999)

| Action | Definition^a^ |
| --- | --- |
| Approach | The emitter comes within one meter of another individual |
| Displacement | After getting within one meter of another individual, the emitter moves back but remains within three meters. |
| Follow | The emitter walks while maintaining close proximity to another individual, ensuring no physical contact. |
| Grooming | The emitter uses one hand to move the partner’s hair aside, while the other hand inspected and cleaned the exposed skin, sometimes using the mouth to remove scabs, parasites, or foreign material. |
| Leave | After being within one meter of another individual, the emitter moves away. |
| Mount | The emitter places its pelvis against the individual's behind |
| Move limb away | The emitter withdraws a limb from another individual |
| Position other | The emitter moves the recipient's body gently, without pushing or pulling, |
| Pull | The emitter seizes and tugs an object or another individual by bending the arms to apply force. |
| Push | The emitter applies force by extending the arms while in contact, causing the recipient to move. |
| Reposition | The emitter changes body position. For example, makes a body part more accessible by either sitting down or standing up. |
| Sniff | The emitter puts its nose close to another individual body |
| Turn head away | The emitter averts its face from another individual |

**Table SM 2** Coded vocalizations along with their definitions ^a^(Range & Fischer, 2004)

| Vocalization | Definition ^a^ |
| --- | --- |
| Copulation call | Vocalization that can last up to ten seconds, occurring primarily during copulation but occasionally also during defecation. |
| Grunt | Soft, low-pitched vocalization produced across different contexts. |
| Twitter | Vocalization ranging from gentle, melodic tones to stronger, almost harsh sounds. |

**
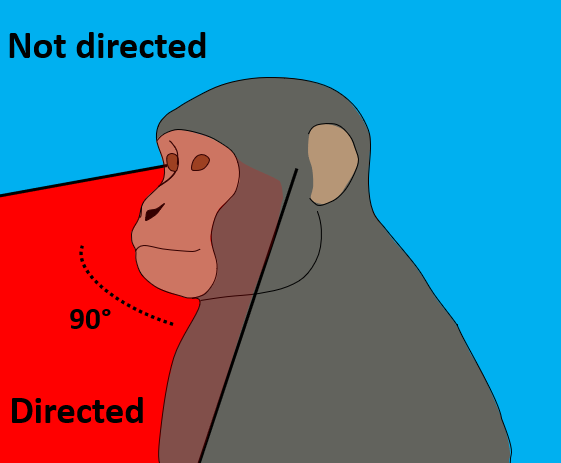
**

**Fig. SM 1** Parameter gaze direction. Directed gaze is defined as the 90 degrees region in front of the individual, measured relative to the midline of the head (45 degrees to the left and 45 degrees to the right). Any gaze within this 90 degrees frontal region is classified as “directed,” while gaze outside this area is classified as “not directed” (Abreu & Pika, 2022; Pika et al., 2003; van Boekholt & Pika, 2025; ; Vlaeyen et al., submitted)


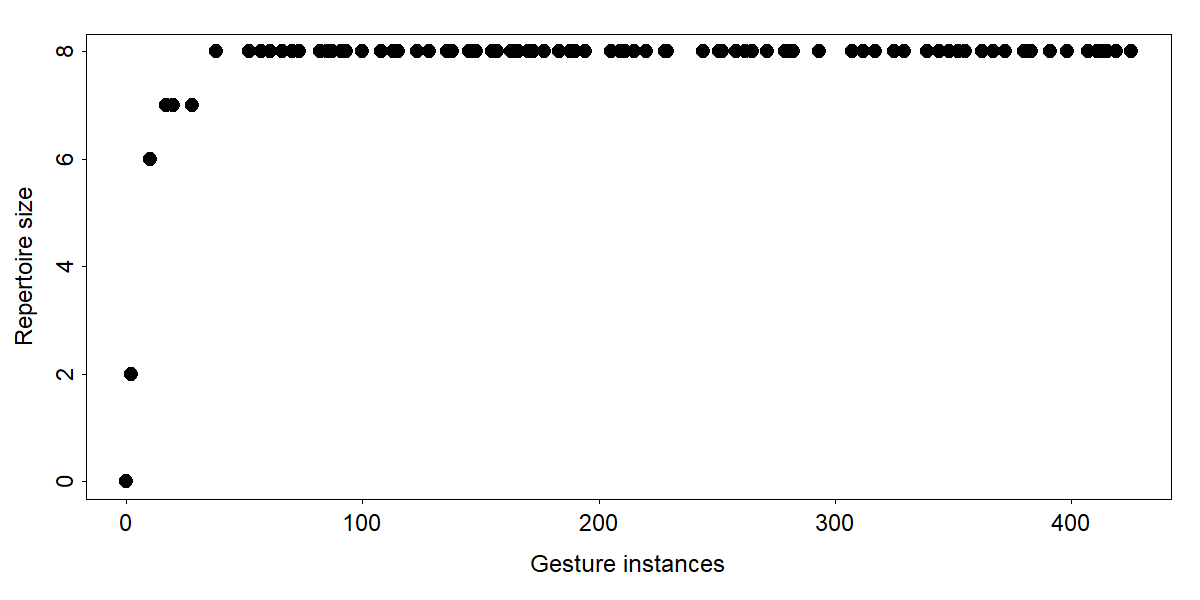


**Fig. SM 2** Size of the gestural repertoire as the number of observed gestures increases (Fröhlich et al., 2016; Genty et al., 2009; Hobaiter & Byrne, 2011). The curve reaches an asymptote, indicating that the full repertoire has been observed


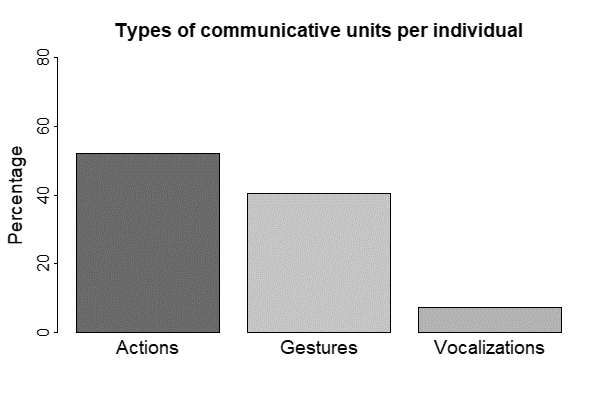


**Fig. SM 3 D**istribution of communicative units, with actions making up 57.15% of the units, gestures accounting for 36.65%, and vocalizations comprising 6.20% of the units per dyad (N=557, N=426, N=92, respectively)

**Fig. SM 4 P**ercentage distribution of turn-transition types per dyad. The average proportion of turn transitions per dyad was 13.12 ± 21.61% for action-action, 27.56 ± 18.34% for action-signal, 38.75 ± 22.49% for signal-action, and 13.53 ± 17.96% for signal-signal turn transitions. A: action, S: signal


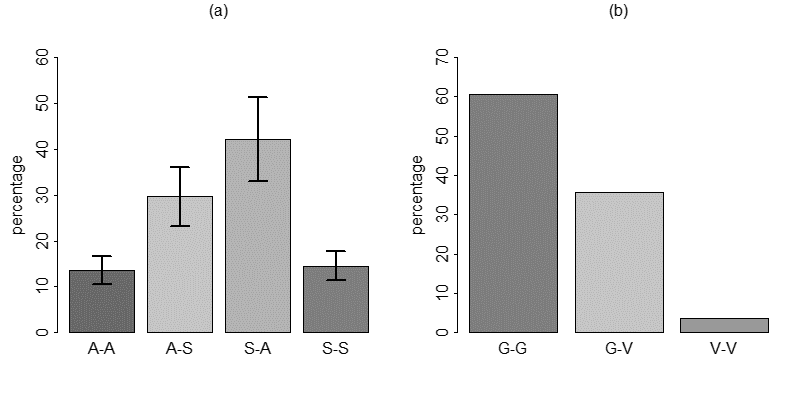


**Fig. SM 5 Shows** that 60.71% of the signal-signal turn transitions were gestural, 35.71% were multimodal (gesture and vocalization), and 3.57% were vocal. G: gesture, V: vocalization

**Table SM 3** List of turn transitions which were associated at a rate higher than expected by chance and observed at least three times along with their corresponding pbin values (pbins > 3 = P < 0.001, > 2 = P < 0.01, and > 1.3 = P < 0.05)

| First unit | Second unit | Pbin |
| --- | --- | --- |
| embrace | embrace | 6.892 |
| Leave | Follow | 5.809 |
| extend limb | Grooming | 1.848 |
| present | Grooming | 27.03 |
| raise | Grooming | 2.673 |
| extend limb | maintain contact | 1.484 |
| Reposition | maintain contact | 2.52 |
| maintain contact | Leave | 1.709 |
| pull | Leave | 1.536 |
| Grooming | extend limb | 4.047 |
| Grooming | present | 19.138 |
| Grunt | raise | 1.544 |
| push | raise | 1.538 |
| pull | Reposition | 6.069 |
| push | Reposition | 4.699 |
| present | Sniff | 1.494 |

**
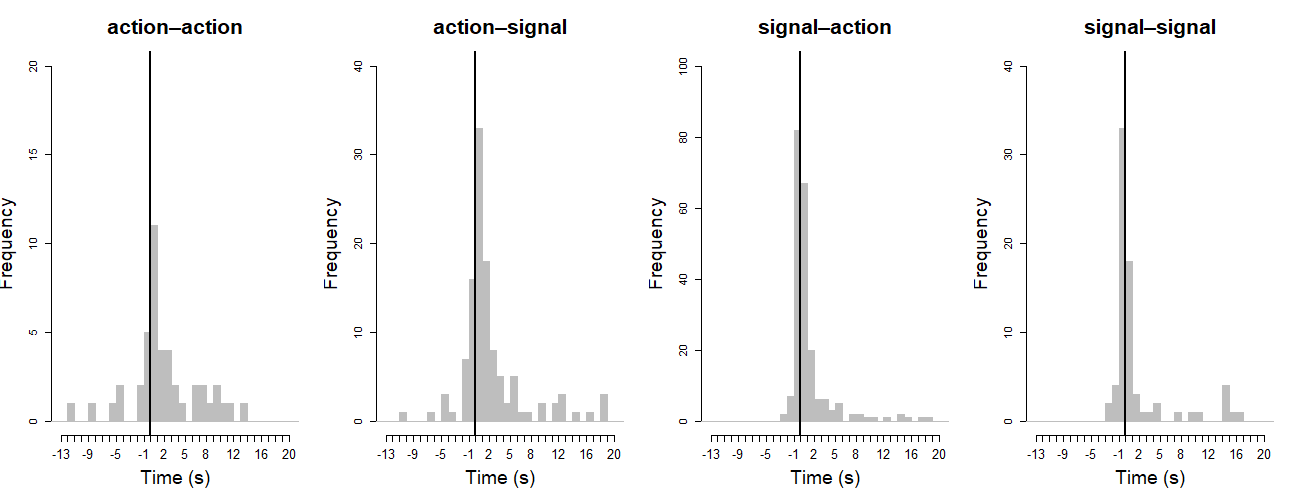
**

**Fig. SM 6 Overview of offset-to-onset temporal relations between turns across the four types of turn transitions. For improved readability, the plot is restricted to a time range of –13 to 20 s.** **A vertical line at 0 s distinguishes overlapping from non-overlapping intervals.**

**
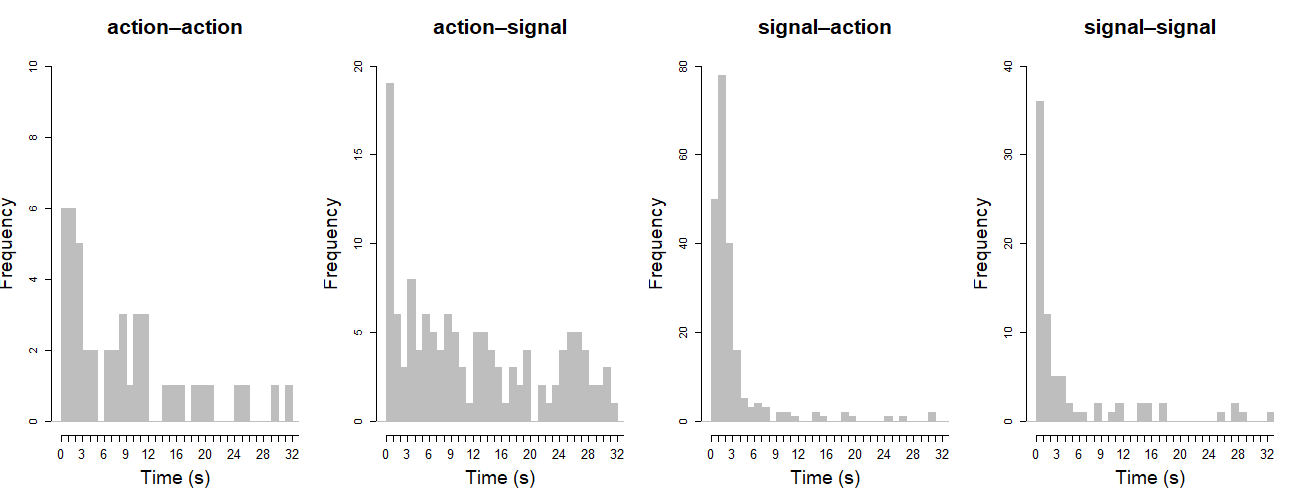
**

**Fig. SM7 Overview of onset-to-onset temporal relations between turns across the four types of turn transitions. For improved readability, the plot is restricted to a time range of 0 s to 32 s.**

**References**

Abreu, F., & Pika, S. (2022). Turn-taking skills in mammals: A systematic review into development and acquisition. *Frontiers in Ecology and Evolution*, *10*, 1168. https://doi.org/10.3389/FEVO.2022.987253/BIBTEX

Fröhlich, M., Wittig, R. M., & Pika, S. (2016). Should I stay or should I go? Initiation of joint travel in mother-infant dyads of two chimpanzee communities in the wild. *Animal Cognition*, *19*(3), 483–500. https://doi.org/10.1007/S10071-015-0948-Z

Genty, E., Breuer, T., Hobaiter, C., & Byrne, R. W. (2009). Gestural communication of the gorilla (Gorilla gorilla): Repertoire, intentionality and possible origins. *Animal Cognition*, *12*(3), 527–546. https://doi.org/10.1007/S10071-009-0213-4/TABLES/5

Hobaiter, C., & Byrne, R. W. (2011). The gestural repertoire of the wild chimpanzee. *Animal Cognition*, *14*(5), 745–767. https://doi.org/10.1007/S10071-011-0409-2/FIGURES/6

Hobaiter, C., & Byrne, R. W. (2014). The Meanings of Chimpanzee Gestures. *Current Biology*, *24*(14), 1596–1600. https://doi.org/10.1016/J.CUB.2014.05.066

Nishida, T., Kano, T., Goodall, J., McGrew, W. C., & Nakamura, M. (1999). Ethogram and Ethnography of Mahale Chimpanzees. *Anthropological Science*, *107*(2), 141–188. https://doi.org/10.1537/ASE.107.141

Pika, S., Liebal, K., & Tomasello, M. (2003). Gestural communication in young gorillas (Gorilla gorilla): Gestural repertoire, learning, and use. *American Journal of Primatology*, *60*(3), 95–111. https://doi.org/10.1002/AJP.10097

Range, F., & Fischer, J. (2004). Vocal Repertoire of Sooty Mangabeys (Cercocebus torquatus atys) in the Taï National Park. *Ethology*, *110*(4), 301–321. https://doi.org/10.1111/J.1439-0310.2004.00973.X

van Boekholt, B., & Pika, S. (2025). Infrastructure of mother-infant interactions across development in chimpanzees (Pan troglodytes) in the wild. *Evolution and Human Behavior*, *46*(2), 106671. https://doi.org/10.1016/J.EVOLHUMBEHAV.2025.106671
