# Supplementary material for: Turn-taking in grooming interactions of sooty mangabeys (Cercocebus atys) in the wild
Source: Anim Cogn. 2026 Mar 10;29(1):40. doi: 10.1007/s10071-025-02040-2 (PMC13102903; doi:10.1007/s10071-025-02040-2)
Supplement: Supplementary file 2 — Supplementary file2 (PDF 53 KB) [file 10071_2025_2040_MOESM2_ESM.pdf]

| Action | Definition[a] |
|---|---|
| Approach | The emitter comes within one meter to another individual |
| Displacement | After getting within one meter of another individual, the emitter moves back but remain within three meters. |
| Follow | The emitter walks while maintaining close proximity to another individual, ensuring no physical contact. |
| Grooming | The emitter uses both hands to push the hair back with the thumb or index finger of one hand, while holding it back and picking at the exposed skin with the nail of the thumb or index finger of the other hand. |
| Leave | After being within one meter of another individual, the emitter moves away. |
| Mount | The emitter places its pelvis against individual's behind |
| Move limb away | The emitter withdraws a limb from another individual |
| Position other | The emitter moves the recipient's body gently, without pushing or pulling, |
| Pull | The emitter seizes and tugs an object or another individual by bending the arms to apply force. |
| Push | The emitter applies force by extending the arms while in contact, causing the recipient to move. |
| Reposition | The emitter changes body position. For example, makes a body part more accessible by either sitting down or standing up. |
| Sniff | The emitter puts nose close to another individual body |
| Turn head away | The emitter averts its face from another individual |
