# Supplementary material for: Turn-taking in grooming interactions of sooty mangabeys (Cercocebus atys) in the wild
Source: Anim Cogn. 2026 Mar 10;29(1):40. doi: 10.1007/s10071-025-02040-2 (PMC13102903; doi:10.1007/s10071-025-02040-2)
Supplement: Supplementary file 3 — Supplementary file3 (PDF 8 KB) [file 10071_2025_2040_MOESM3_ESM.pdf]

Vocalization	Definition [a]
Copulation call	Vocalization that can last up to ten seconds, occurring primarily during copulation but occasionally also during defecation.
Grunt	Soft, low-pitched vocalization produced across different contexts.
Twitter	Vocalization ranging from gentle, melodic tones to stronger, almost harsh sounds.
