# Supplementary material for: Turn-taking in grooming interactions of sooty mangabeys (Cercocebus atys) in the wild
Source: Anim Cogn. 2026 Mar 10;29(1):40. doi: 10.1007/s10071-025-02040-2 (PMC13102903; doi:10.1007/s10071-025-02040-2)
Supplement: Supplementary file 4 — Supplementary file4 (PDF 64 KB) [file 10071_2025_2040_MOESM4_ESM.pdf]

First unit	Second unit	Pbin
EMBRACE	EMBRACE	6.892
Leave	Follow	5.809
EXTEND LIMB	Grooming	1.848
PRESENT	Grooming	27.03
RAISE	Grooming	2.673
EXTEND LIMB	MAINTAIN CONTACT	1.484
Reposition	MAINTAIN CONTACT	2.52
MAINTAIN CONTACT	Leave	1.709
PULL	Leave	1.536
Grooming	EXTEND LIMB	4.047
Grooming	PRESENT	19.138
Grunt	RAISE	1.544
PUSH	RAISE	1.538
PULL	Reposition	6.069
PUSH	Reposition	4.699
PRESENT	Sniff	1.494
